# Coronary Artery Disease in Very Young Patients: Analysis of Risk Factors and Long-Term Follow-Up

**DOI:** 10.3390/jcdd9030082

**Published:** 2022-03-11

**Authors:** Pablo Juan-Salvadores, Víctor Alfonso Jiménez Díaz, Cristina Iglesia Carreño, Alba Guitián González, Cesar Veiga, Cristina Martínez Reglero, José Antonio Baz Alonso, Francisco Caamaño Isorna, Andrés Iñiguez Romo

**Affiliations:** 1 Cardiovascular Research Unit, Cardiology Department, Hospital Alvaro Cunqueiro, University Hospital of Vigo, 36213 Vigo, Spain; victor.alfonso.jimenez.diaz@sergas.es; 2 Cardiovascular Research Group, Galicia Sur Health Research Institute (IIS Galicia Sur), Servizo Galego de Saude, Universidade de Vigo, 36213 Vigo, Spain; cesar.veiga@iisgaliciasur.es (C.V.); jose.baz.alonso@sergas.es (J.A.B.A.); andres.iniguez.romo@sergas.es (A.I.R.); 3 Interventional Cardiology Unit, Cardiology Department, Hospital Álvaro Cunqueiro, University Hospital of Vigo, 36213 Vigo, Spain; 4 Cardiology Department, Hospital Álvaro Cunqueiro, University Hospital of Vigo, 36213 Vigo, Spain; cristina.victoria.iglesia.carreno@sergas.es (C.I.C.); alba.guitian.gonzalez@sergas.es (A.G.G.); 5 Methodology and Statistics Unit, Galicia Sur Health Research Institute (IIS Galicia Sur), Servizo Galego de Saude, Universidade de Vigo, 36213 Vigo, Spain; cristinamreglero@gmail.com; 6 Department of Preventive Medicine, University of Santiago de Compostela, 15782 Santiago de Compostela, Spain; francisco.caamano@usc.es; 7 Consortium for Biomedical Research in Epidemiology and Public Health (CIBER en Epidemiología y Salud Pública-CIBERESP), 15782 Santiago de Compostela, Spain

**Keywords:** coronary artery disease, risk factors, young, percutaneous coronary intervention, clinical epidemiology, acute coronary syndrome, stable angina

## Abstract

Coronary artery disease (CAD) is a common chronic condition in the elderly. However, the earlier CAD begins, the stronger its impact on lifestyle and costs of health and social care. The present study analyzes clinical and angiographic features and the outcome of very young patients undergoing coronary angiography due to suspected CAD, including a nested case-control study of ≤40-year-old patients referred for coronary angiography. Patients were divided into two groups: cases with significant angiographic stenosis, and controls with non-significant stenosis. Of the 19,321 coronary angiographies performed in our center in a period of 10 years, 504 (2.6%) were in patients ≤40 years. The most common cardiovascular risk factors for significant CAD were smoking (OR 2.96; 95% CI 1.65–5.37), dyslipidemia (OR 2.18; 95% CI 1.27–3.82), and family history of CAD (OR 1.95; 95% CI 1.05–3.75). The incidence of major adverse cardiovascular events (MACE) at follow-up was significantly higher in the cases compared to controls (HR 2.71; 95% CI 1.44–5.11). Three conventional coronary risk factors were directly related to the early signs of CAD. MACE in the long-term follow-up is associated to dyslipidaemia and hypertriglyceridemia. Focusing efforts for the adequate control of CAD in young patients is a priority given the high socio-medical cost that this disease entails to society.

## 1. Introduction

Coronary artery disease (CAD) is a chronic condition usually occurring after the sixth decade of life [1]. However, some populations experience this disease prematurely [2]. In the last few decades, different studies have shown an increased incidence of CAD in very young people [3,4,5], mainly in Asian populations [6]. Even though they are relatively few in number, these young patients with CAD represent a significant economic and health care burden for society, thus becoming chronic patients [7]. Hence, focusing research efforts on proper control is a priority. Histological analyses have shown the presence of subclinical atheromatous plaques in earlier stages of life [8,9], which may represent future substrates for the prompt onset of significant coronary stenosis.

The prevalence of CAD reported in very young people is 1–16% [10,11,12,13,14], and acute coronary syndrome (ACS) is the most common clinical presentation. Nevertheless, the results of the coronary angiography performed due to suspected CAD displayed normal coronary arteries or those without significant lesions in a large number of young patients [15].

The scientific evidence about the risk factors and long-term prognosis that predispose patients towards early CAD is currently scarce [16]. Although several population studies have reported a correlation with classical risk factors [12,17,18], the differences in the cut-off point of age and the definition of the disease limit their power to identify the triggering factors for early CAD and its long-term prognostic implications. Additionally, few data exist on the prognosis of subjects under the age of 40 with cardiovascular risk factors and normal coronary arteries or with non-significant coronary stenosis detected by coronary angiography [19,20].

This study analyses the relevance of cardiovascular risk factors in very young patients presenting CAD, as well as their possibility of experiencing major adverse cardiovascular events (MACE).

## 2. Materials and Methods

### 2.1. Design and Study Population

This single-center, retrospective, nested case-control study in a cohort of patients ≤40 years old referred for the first time to coronary angiography due to clinical suspicion (electrocardiographic changes, biomarkers of myocardial injury, or a positive ischemia stress test) of CAD, including ACS or stable angina.

**Figure 1:**
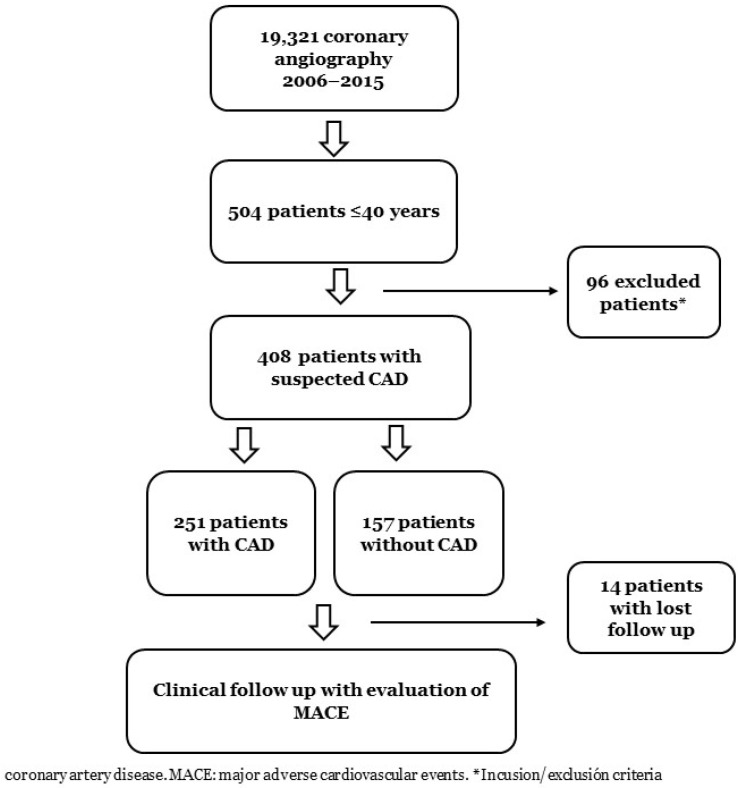
Flow chart diagram of the study population.

From 1 January 2006 to 31 December 2015, a total of 19,321 coronary angiograms were performed in our hospital, and 504 (2.6%) were in patients ≤40 years old. Overall, 96 of them were excluded, and 408 (2.1%) patients were ultimately included in the study (Figure 1). We defined as cases (*n* = 251) those with angiographically significant coronary stenosis (≥75% of the luminal diameter determined visually or ≥50% by quantitative coronary analysis) or any positive invasive fractional flow reserve test in one or more epicardial vessels. In the left main, stenosis ≥50% was considered a significant disease. The control group (*n* = 157) was defined as patients with non-significant stenosis (<75%). The detailed inclusion and exclusion criteria were as follows.

Cases inclusion criteria: Women and men aged between 18 and 40 years. Referred for coronary angiography due to signs or symptoms suggestive of CAD, or a positive ischemia stress test. Presenting at least one angiographically significant coronary stenosis.

Controls inclusion criteria: The same as above, but with absence of significant angiographic coronary stenosis.

Cases and controls exclusion criteria: Patients with prior coronary revascularization (percutaneous or surgical). Referral for coronary angiography for any other non-ischemic cause (pre-transplant, pulmonary hypertension, structural disease, or others).

This study restricted the inclusion to a population aged ≤40 since the risk of suffering a coronary ischemic event increases significantly in men from 40 years of age, as reported by the Coronary Artery Risk Development in Young Adults Study (CARDIA) group, which described a difference in prevalence of 13.3% vs. 5.5% among patients aged 40–45 years and 33–39 years, respectively [21]. 

### 2.2. Data Collection and Definition of Variables

The study population came from a tertiary hospital, a reference center for cardiovascular disease. The information was obtained through the specific database of the Interventional Cardiology Unit, disaggregated by personal data. The follow-up was conducted by database analysis, which was updated by electronic medical records.

The variables were defined as: arterial hypertension (defined as a previous medical diagnosis recorded in the clinical history or by the use of hypertensive medication), diabetes mellitus (existence of a previous diagnosis or the use of hypoglycemic medication), dyslipidemia (existence prior diagnosis or use of lipid-lowering medication), body mass index (BMI) (classified as normal weight <25, overweight 25–29, and obesity >30), smoking (current or former smoker), drug use (active of toxic substances recognized by the patient or by laboratory tests), chronic renal failure (existence of a previous diagnosis or being under chronic hemodialysis treatment), peripheral arterial disease (existence of documented history of peripheral arterial disease, claudication, amputation due to arterial insufficiency, occlusive aortoiliac disease, or inter surgical or percutaneous peripheral arterial revascularization) and prior stroke documented in medical history, family history of coronary artery disease (defined as direct family history of cardiovascular disease before age 55 for men, and before age 65 for women), atrial fibrillation (considered as a history of a previous diagnosis or documented ECG), depression (considered as a history of a previous diagnosis or use of antidepressant medication), and congestive heart failure (considered as a history of a previous diagnosis). The MACE definition included death, myocardial infarction, stroke, and new coronary revascularizations (the appearance of one of these events for the first time in each patient).

### 2.3. Ethical and Legal Aspects

The investigators participating in this study followed the applicable ethical and legal standards. This study was approved by the Regional Research Ethics Committee with registration code 2015/506.

### 2.4. Statistical Analysis

Descriptive statistics are reported as mean± standard deviation (SD), median with interquartile range (IQR) for continuous variables, and numbers and percentages for categorical variables. A univariable analysis was performed to detect significant differences between the two groups for the primary variables using the Fisher exact test, χ^2^ test, Student *t* test, or Mann–Whitney U test, as appropriate. To determine the risk combination contribution, a multivariate model was used using binary logistic regression analysis. The cardiovascular risk factors analyzed are given as odds ratios (OR) together with their 95% confidence intervals. Kaplan–Meier curves and the log-rank test were used to compare the time to the occurrence of a combination of events between the two groups of patients. To identify the factors associated with the combination of events tested, a multivariate Cox regression analysis was performed, calculating the hazard ratio (HR) and 95% confidence intervals. To evaluate recurrent events, the Andersen and Gill model was used. For the data analysis, the SPSS program for Windows, version 19, was used. Likewise, the R program, version 3.6.1, and the survival packages for the Andersen and Gill model and finalfit for the figures were used.

## 3. Results

The admission clinical characteristics of the patients are shown in Table 1. Age had an asymmetric distribution for all study subjects, with a median of 37 years and an interquartile range (IQR) of 34–39 years. Age was higher in the case group (median 37 years, IQR 34–39 years) compared to the control group (median 36 years, IQR 32–38 years; *p* = 0.011). Male gender predominated in the case group, with higher levels of total cholesterol, low-density lipoproteins (LDL), and lower levels of high-density lipoproteins (HDL) compared to the controls. The most common clinical presentation was ACS, with 232 (92.4%) in the case group and 98 (62.4%) in the control group. Patients with multivessel disease were detected in 66 (26.3%) cases. Angiographic and procedural characteristics are listed in Appendix A as Table A1.

**Figure 2:**
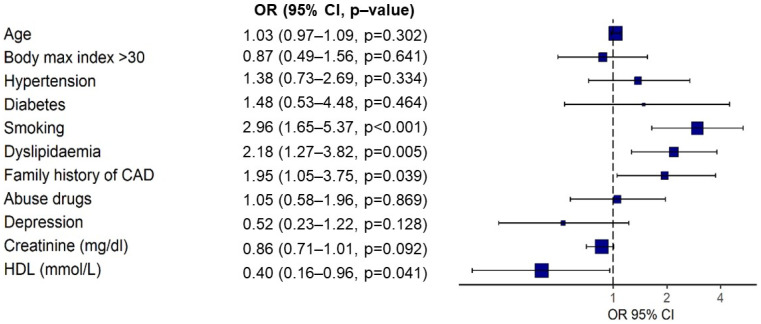
Multivariate logistic regression analysis of risk factors associated with significant CAD. CAD: coronary artery disease. HDL: high-density lipoprotein.

The multivariate logistic regression analysis showed that smoking, dyslipidemia, and a family history of CAD were significantly associated with the appearance of early CAD, and high HDL levels were shown to be a protective factor for early CAD (Figure 2).

In the case group, the indication of coronary angiography is the same as that of the final diagnosis at hospital discharge (Table 1). The most frequent treatment (86%) was percutaneous revascularization followed by non-invasive pharmacological treatment (9.6%) and surgical revascularization (4.4%). 

In the control group, the most common final diagnosis was non-cardiac chest pain in 57 patients (36.3%), myocardial infarction with no obstructive coronary atherosclerosis (MINOCA) in 32 patients (20.4%), pericarditis in 31 patients (19.7%), myocarditis in 28 patients (17.8%), and others in 9 patients (5.8%). 

**Figure 3:**
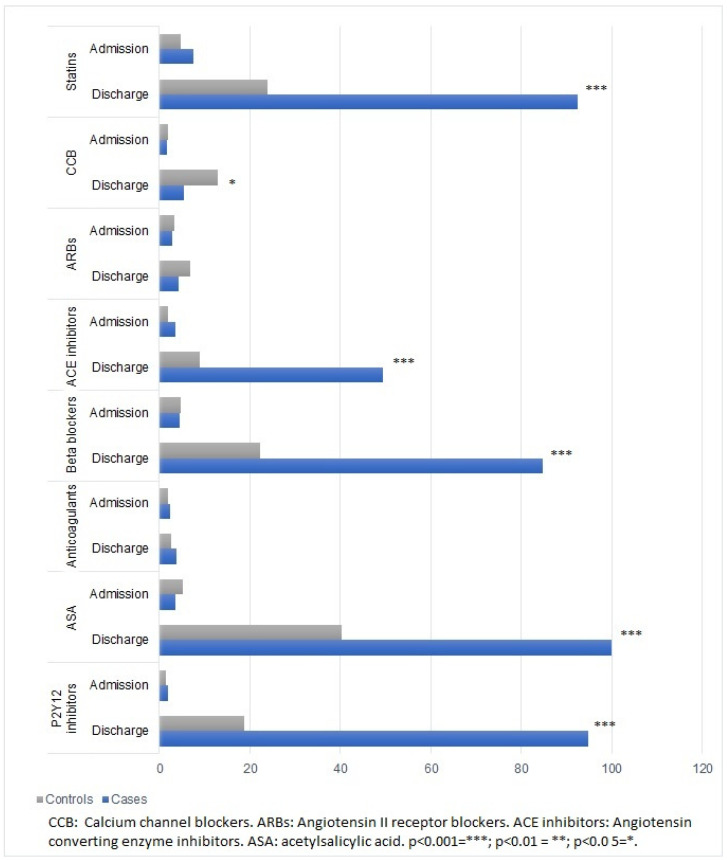
Pharmacological treatment at hospital admission and at discharge.

The pharmacological treatment of both groups is shown in Figure 3. On admission, no significant differences were found in relation to pharmacological treatment between groups. At discharge, statistically significant differences were found between cases and controls in relation to statins, ACE inhibitors, beta-blockers, and antiplatelet therapy. On the other hand, calcium channel blockers were more often prescribed in the control group. 

The mean follow-up time was 4.98 ± 2.1 years (minimum 1 year, maximum of 9 years) in the case group. In the control group, the mean follow-up time was 5.34 ± 2.3 year (minimum 1 year, maximum of 9 years). Clinical follow-up was not completed in 14 subjects (3.4%). A total of 70 events were detected, mainly due to the need for new coronary revascularizations. Additionally, new target lesion revascularization was detected in 13 cases. The case group shows a higher incidence of MACE during the follow-up compared to the control group (Table 2).

**Figure 4:**
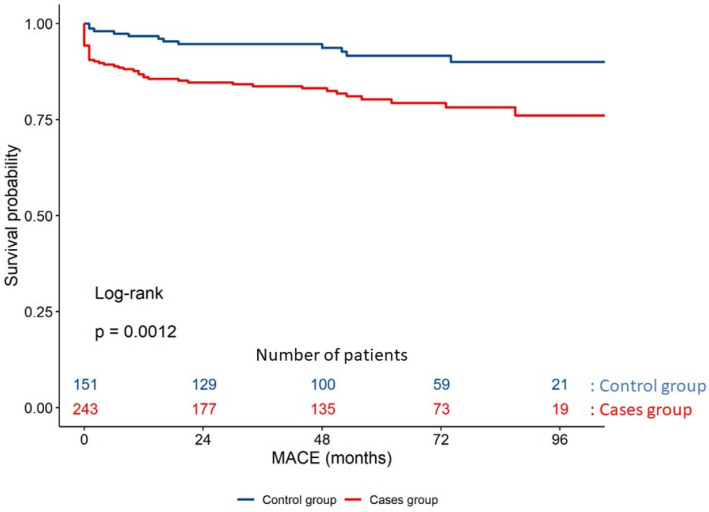
Clinical outcomes of case and control groups at the time of the first MACE.

**Figure 5:**
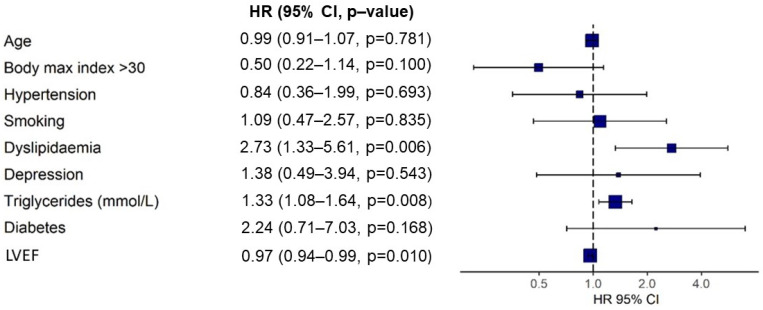
Cox regression model of risk factors associated with MACE at follow-up. LVEF: Left ventricular ejection fraction.

The survival function for MACE is displayed in Figure 4. The analysis of the association between MACE and the different cardiovascular risk factors is shown in Figure 5; this study showed that the presence of dyslipidemia and hypertriglyceridemia increases the risk of MACE at long-term follow-up. 

In order to evaluate recurrent events, the model revealed that the risk for MACE recurrence triples annually in the case group (HR 3.15, 95% CI 1.71–5.81; *p* < 0.001) compared to the control group. The risk factors associated with an increased risk of recurrent MACE are dyslipidemia (HR 2.26, 95% CI 1.22–4.20), hypertriglyceridemia (HR 1.20, 95% CI 1.03–1.41), and diabetes (HR 2.28, 95% CI 1.10–4.70). MINOCA patients displayed four times greater risk for MACE recurrence than subjects in the control group (HR 4.13, 95% CI 1.22–13.89).

## 4. Discussion

The main findings of our study can be summarized as follows: First, the presence of smoking, dyslipidemia, and a family history of CAD were associated with the presence of significant CAD in very young patients. Second, patients with dyslipidemia and hypertriglyceridemia have a higher risk of MACE at long-term follow-up. Third, patients in the case group and with MINOCA showed a more unfavorable long-term prognosis compared to the control group. Interestingly, patients with MINOCA showed a similar prognosis to the case group, irrespective of the treatment modality received.

### 4.1. Risk Factors for Early CAD

In young people, CAD seems to be the result of the concurrence and interaction of multiple modifiable and non-modifiable risk factors. Among the modifiable factors, the influence of smoking is particularly relevant for the early appearance of coronary atherosclerosis. The percentage of smokers in our study is among the highest in the literature [22] and is comparable to the 83.6% reported in the PRHIAMO II study in the Spanish population [12]. In our study, smoking increased the risk of suffering significant CAD almost three-fold, even with a restricted time of tobacco exposure in our population, showing the relevance of tobacco in the development of early CAD. 

In our research, the prevalence of dyslipidemia was of more than half of the population affected in the case group, with higher mean total LDL-cholesterol levels and triglycerides than the values reported in different trials analyzing young populations [23]. Dyslipidemia involves more than double the risk of CAD, even though the time of exposure to this factor was limited due to the age restriction in our study. Additionally, in our investigation, the control group showed higher HDL values, granting a protector factor against CAD. The potential role of the Atlantic diet and (highly prevalent in our study population) in lipoprotein levels deserves a detailed analysis. Dyslipidemia may be an underdiagnosed disease in the population ≤40 years of age, as shown in the treatment schemes on hospital admission, with only 19 (7.6%) of the cases having an active prescription of statins [24].

The genetic variations involving a family history of early CAD, together with inherited lifestyle habits [25], might have an impact on the early manifestation of CAD. In our study, the presence of a family history of early CAD increased the risk of CAD almost two-fold [22,26,27]. It seems reasonable to carry out greater healthcare and educational control in the first-degree relatives of these patients.

Diabetes mellitus is a heterogeneous mix of health conditions characterized by glucose dysregulation, and it has an effect on the premature onset and severity of atherosclerosis. Therefore, diabetes is one of the most prevalent risk factors for coronary artery disease in the general population older than 40 years (1); however, in young subjects with CAD, a direct association has not been strongly described. Due the pathophysiology of this disease, it likely requires long periods of time to induce organ damage. 

Other studies have reported mixed results regarding the role of hypertension, diabetes, obesity, and drug abuse as triggering factors of CAD in young patients [11,17,22]. Our study fails to demonstrate a significant relationship between these conventional risk factors and the presence of CAD, but their association is plausible. The low incidence of these diseases in our population and the need for a longer time of exposure might explain our results. It should be noted that the definition of young patient and CAD are heterogeneous in the literature. Furthermore, the control group does not seem to be ideal, which could lead to overestimating the risk of any of these risk factors in the young population [12,14,27]. It is noteworthy that the number of illicit drug and alcohol abuse users is higher in our study than in the general population [28]. These results warn of the high prevalence of users who undergo coronary angiography due to suspected CAD. Therefore, there could be an association between the consumption of illicit drugs and alcohol abuse and the performance of invasive tests to rule out CAD rather than non-invasive diagnostic tests preferentially, which would increase the use of health resources in this population. 

The final diagnosis at hospital discharge is varied in the control group, the most common being non-cardiac chest pain, implying no need for any invasive management. However, it would require lifestyle modifications and, in some cases, pharmacological treatment. A current challenge is to perform a more thorough screening, supported by non-invasive tests for the diagnosis of this condition.

The objective of pharmacological treatment is to reduce anginal symptoms as well as preventing adverse events, representing one of the pillars of secondary prevention. Significant differences have been found between the two study groups, since they are different clinical diagnoses after coronary angiography. An effort must be made by all health professionals to achieve adequate therapeutic adherence, especially in this young population conditioned by low risk appreciation [29]. The evidence generated is clear, since poor adherence increases the risk of suffering an adverse event [30].

### 4.2. Follow-Up

During a mean follow-up time of >5 years, more repeated events were detected for new coronary revascularizations, similar to in other studies [31]. The mortality was low in both groups. In our trial, we found that mortality was higher in the control group in the long-term follow-up (>5 years). The multiple confounders and the fact that the design of this study was not aimed at achieving this objective might be related to this finding. Some studies with a shorter follow-up have reported a mortality rate ranging from 0.2% [24] to 8.5% [17]. Jason et al. reported a 31% mortality at 15 years [31]. Hence, secondary prevention is key in this population.

In our study, non-conventional risk factors for CAD, such as hypertriglyceridemia and left ventricular function, became relevant at follow-up. Patients with high triglyceride levels showed an increased incidence of MACE at follow-up. These data were consistent with our recurrent events model, except for diabetes, which acquires statistical significance for high MACE incidence at follow-up. Focusing efforts on these risk factors could help to achieve a more effective secondary prevention.

MINOCA patients had a similar pattern of pharmacological treatment at hospital discharge to the rest of the patients in the control group, in line with other reports [14,17]. However, in our analysis, this subgroup of patients had a four times higher incidence of MACE than the controls, with worse clinical outcomes compared to patients with significant CAD. The study by Radillis et al. found a better prognosis for these patients than for those with obstructive disease [32]. However, the results of the VIRGO study refer to a similar prognosis for both groups [19]. Therefore, it will be necessary to generate more evidence to confirm the prognosis of these patients. According to our results, this subgroup may require a different therapeutic approach and closer follow-up. The worst prognostic values at follow-up may be affected by the laxity in secondary prevention.

This study has several limitations. First, the presence of non-traditional cardiovascular risk factors, such as lipoprotein A, hyperhomocysteinemia, and familial hypercholesterolemia, was not analyzed. It is likely that the relationship of prothrombotic diseases with the early appearance of ischemic heart disease could not be assessed due to the lack of data in most patients. Second, due to the nature of our study, selection biases cannot be ruled out. Nonetheless, our study includes a consecutive cohort with the largest sample sizes reported in the literature in this group of patients. 

## 5. Conclusions

In our study, current smokers, dyslipidemia, and familial history of CAD were found to be significantly associated with the existence of severe CAD in patients of ≤40 years. The presence of dyslipidemia and hypertriglyceridemia increases the risk of MACE at long-term follow-up. Focusing efforts on primary prevention and optimal control of these factor is necessary. A detailed genetic analysis of high-risk populations with a family history of CAD must be considered. 

## Figures and Tables

**Table 1:** Clinical characteristics of patients ≤40 years undergoing coronary angiography.

Variables	Overall (*n* = 408)	Cases (*n* = 251)	Controls (*n* = 157)	*p*-Value
Women	52 (12.7%)	29 (11.6%)	23 (14.6%)	0.364
Age (median and IQR)	37 (34–39)	37 (34–39)	36 (32–38)	0.011
Follow-up time (years)	5.1 ± 2.2	5.0 ± 2.2	5.3 ± 2.3	0.118
Body max index > 30	127 (31.1%)	82 (32.7%)	45 (28.7%)	0.395
Hypertension	84 (20.6%)	54 (21.5%)	30 (19.1%)	0.559
Diabetes	23 (5.6%)	16 (6.4%)	7 (4.5%)	0.414
Smoking	315 (77.2%)	214 (85.3%)	101 (64.3%)	0.001
Dyslipidaemia	163 (40.0%)	124 (49.4%)	39 (24.8%)	0.001
Family history of CAD	94 (23.0%)	73 (29.1%)	21 (13.4%)	0.001
Illicit drugs and alcohol	91 (22.3%)	56 (22.3%)	35 (22.7%)	0.997
Cannabis	42 (10.3%)	30 (12.0%)	12 (7.6%)	0.163
Opioids	8 (2.0%)	4 (1.6%)	4 (2.5%)	0.491
Alcohol	43 (10.5%)	26 (10.4%)	17 (10.8%)	0.881
Cocaine	43 (10.5%)	28 (11.2%)	15 (9.6%)	0.608
Peripheral artery disease	4 (1.0%)	3 (1.2%)	1 (0.6%)	0.999
Congestive heart failure	2 (0.5%)	1 (0.4%)	1 (0.6%)	0.999
Previous stroke	3 (0.7%)	2 (0.8%)	1 (0.6%)	0.999
Atrial fibrillation	3 (0.7%)	2 (0.8%)	1 (0.6%)	0.999
Renal failure	16 (3.9%)	6 (2.4%)	10 (6.4%)	0.044
Depression	32 (8.2%)	17 (6.8%)	15 (9.6%)	0.309
Total cholesterol (mmol/L)	5.06 ± 1.3	5.25 ± 1.4	4.72 ± 1.0	0.001
LDL cholesterol (mmol/L)	3.25 ± 1.2	3.42 ± 1.3	2.91 ± 0.8	0.001
HDL cholesterol (mmol/L)	1.03 ± 0.3	0.99 ± 0.3	1.09 ± 1.4	0.002
Triglycerides (mmol/L)	1.77 ± 1.1	1.831 ± 1.2	1.69 ± 0.9	0.240
Creatinine (mg/dL)	1.2 ± 1.6	1.0 ± 1.1	1.4 ± 2.1	0.081
Glucose (mmol/L)	5.8 ± 2.3	5.96 ± 2.3	5.68 ± 2.3	0.269
LVEF (%)	55 ± 9.5	54 ± 8	57 ± 10	0.034
Admission Symptoms				
Chest pain	370 (91.1%)	240 (95.6%)	130 (83.9%)	0.001
Dyspnea	32 (7.9%)	17 (6.8%)	15 (9.7%)	0.344
Shock	16 (3.9%)	12 (4.8%)	4 (2.6%)	0.307
Coronary angiography indication				
ACS	330 (80.9%)	232 (92.4%)	98 (62.4%)	0.001
STEMI	195 (59.1%)	150 (64.7%)	45 (45.9%)	
NSTEMI	135 (40.9%)	82 (35.3%)	53 (54.1%)	
Stable angina	65 (15.9%)	17 (6.8%)	48 (30.6%)	
Asymptomatic with positive for ischemia detection	13 (3.2%)	2 (0.8%)	11 (7.0%)	
Hospitalization days	6.6 ± 7.7	7.2 ± 7.3	5.6 ± 8	0.044

Data are given as number (percentage) or mean ± SD. ACS: acute coronary syndrome. CAD: coronary artery disease. HDL: high-density lipoproteins. LDL: low-density lipoproteins. LVEF: left ventricle ejection fraction. NSTEMI: non-ST-elevation myocardial infarction. STEMI: ST-elevation myocardial Infarction.

**Table 2:** Adverse events in the study period.

Adverse Events	Overall (*n* = 394)	Cases (*n* = 243)	Controls (*n* = 151)	*p*-Value	HR CI 95%
New coronary revascularizations	40 (10.1%)	34 (14.0%)	6 (4.0%)	0.001	4.34 (1.81–10.39)
Death	14 (3.5%)	8 (3.0%)	6 (4.0%)	0.805	0.87 (0.30–2.52)
AMI	10 (2.5%)	8 (3.3%)	2 (1.3%)	0.227	2.52 (0.53–11.86)
Stroke	6 (1.5%)	5 (2.1%)	1 (0.7%)	0.262	3.42 (0.40–29.30)
MACE	59 (14.9%)	47 (19.3%)	12 (7.9%)	0.002	2.71 (1.44–5.11)

Data are given as number (percentage). AMI: acute myocardial infarction. MACE; major adverse cardiovascular events, death, acute myocardial infarction, stroke, and new coronary revascularizations.

## Data Availability

The data used to support the findings of this study are available from the corresponding author upon reasonable request.

## Appendix A

**Table A1:** Angiographic characteristics of patients with CAD.

Variables	Cases (251)
General Characteristics	
Number of vessels with significant stenosis	1.41 ± 0.82
Number of treated lesions	1.15 ± 0.76
Number of treated vessels	1.02 ± 0.51
Implanted stents	1.07 ± 0.77
Culprit Lesion	
Affected coronary artery	
Left anterior descending and branches	130 (51.8%)
Circumflex and branches	38 (15.1%)
Right coronary and branches	77 (30.7%)
Left main	6 (2.4%)
Baseline stenosis (%)	92.9 ± 8.9
Baseline TIMI flow	
0	109 (43.8%)
1	22 (8.8%)
2	26 (10.4%)
3	92 (36.9%)
AHA lesion classification	
Type A	21 (10.8%)
Type B1	38 (19.0%)
Type B2	57 (28.5%)
Type C	84 (42.0%)
Thrombectomy	92 (38.7%)
Stent diameter (mm)	3.26 ± 0.5
Stent length (mm)	20.27 ± 8.2
BMS	107 (50.5%)
BVS	13 (6.1%)
DES	92 (43.4%)
Paclitaxel	11 (11.9%)
Limus family drugs	81 (88.1%)
Final TIMI flow	
0	24 (9.7%)
1	0 (0%)
2	2 (0.8%)
3	221 (89.5%)
Final stenosis (%)	9.34 ± 26.8

Data are given as number (percentage) or mean ± SD. AHA: American Heart Association. BMS: bare metal stent. BVS: bioresorbable vascular scaffold. DES: drug eluding stent. TIMI: thrombolysis in myocardial infarction.
